# Selective Growth Inhibitory Effect of Biochanin A Against Intestinal Tract Colonizing Bacteria

**DOI:** 10.3390/molecules15031270

**Published:** 2010-03-03

**Authors:** Olga Sklenickova, Jaroslav Flesar, Ladislav Kokoska, Eva Vlkova, Katerina Halamova, Jan Malik

**Affiliations:** 1 Department of Crop Sciences and Agroforestry, Institute of Tropics and Subtropics, Czech University of Life Sciences Prague, Kamycka 129, Prague 6, 165 21, Czech Republic; E-Mails: sklenickova@its.czu.cz (O.S.); halamova@its.czu.cz (K.H.); malikj@its.czu.cz (J.M.); 2 Department of Microbiology, Nutrition and Dietetics, Faculty of Agrobiology, Food and Natural Resources, Czech University of Life Sciences Prague, Kamycka 129, Prague 6, 165 21, Czech Republic; E-Mails: flesar@af.czu.cz (J.F.); vlkova@af.czu.cz (E.V.)

**Keywords:** phytoestrogen, isoflavonoid, bifidobacteria, clostridia, selective antimicrobial activity

## Abstract

Both bifidobacteria and clostridia are part of the natural gut microflora and while clostridia may be responsible for severe intestinal infections, bifidobacteria are probiotic microorganisms belonging to the most important prospective bacteria in the bowel. The antimicrobial activity of biochanin A was tested *in vitro* against six *Bifidobacterium* spp., and eight *Clostridium* spp. using the broth microdilution method. Biochanin A showed an inhibition against all clostridia in the range of minimum inhibitory concentrations (MIC) from 64 μg/mL (for *Cl. clostridioforme*, strains DSM 933 and I3) to 1,024 μg/mL (for *Cl. perfringens*, DSM 11778). No bifidobacteria were suppressed at four-fold higher concentration (MICs > 4,096) than MIC of *Cl. perfringens*. These results indicate selective growth inhibition of biochanin A and its potential use in antimicrobial prevention and/or protection.

## 1. Introduction

Intestinal infections are extremely frequent disorders occurring in humans as well as in animals [1]. They are either the result of pathogens or a pathological overgrowth of an opportunistic member of the intestinal microecosystem. Microbial infections manifesting themselves as diarrhoea, fever, vomiting and nausea are mostly induced by enteropathogenic microorganisms like toxigenic *E. coli*, shigellae and salmonellae, yersiniae, campylobacteria and clostridia [1,2]. Among gastrointestinal diseases, *Clostridium*-associated diseases (CADs) have assumed worrisome proportions because of their enormous medical and economic impact [3]. Clostridia naturally reside in limited amounts among intestinal microflora [4]. When overpopulated, they may be responsible for serious infections like pseudomembranous colitis and antibiotic-associated diarrhoea caused by *Cl. difficile* [5], inflammation by enterotoxin or cytotoxin from *Cl. perfringens* [6] or secondary infections due to *Cl. ramosum* [7,8] and *Cl. clostridioforme* [9,10,11]. The most statistically prevalent species is *Cl. difficile*. It was reported to be a major cause of infectious diarrhoea peaking at 21 per 1000 hospitalized patients in Dublin (Ireland, 2003-2004) [12] and to cause a mortality rate of 23.7 per million admissions in the USA (2004) [13]. Kuijper *et al*. [3] estimated the financial impact of only the *Cl. difficile* on the healthcare system to be as high as 3 billion €/year of total expenditures in European Union and $1.1 billion/year the USA. This is expected to almost double over the next four decades.

Broad-spectrum antibiotics (ATB), particularly those with activity against anaerobes, are used to treat CADs [8,14,15]. Metronidazole, tetracycline and fluoroquinolones remain the first-line therapy with vancomycin as a reserve for the most severe cases [8,14,15,16,17]. However, from 20% to 30% of incidents go on to experience further episodes several days after the ATB is discontinued. When ATB fails during the initial treatment cycle, there is a high risk of recurrence after cessation of the medication [15,18,19,20]. The search for a new ATB treatment may not be the most effective strategy for treating CADs because most ATB’s share the risk of ATB-resistance [21], and can perturb the gut microbiota [22], which is a well-balanced ecosystem belonging to the immune complex [4]. The periodical disturbances of co-existing microflora evoked by ATB often result in further infections which can create a vicious circle. Among the protective microbiota in guts, *Bifidobacterium* species have been recognized as probiotic organisms; they play a crucial role in maintaining gastrointestinal health, promote digestion, boost the immune system [23] and inhibit the growth of certain microorganisms [24] including clostridia [25,26]. The harmful incidence of ATB erases the positive effects of bifidobacteria, alters the gut microflora, disrupting its barrier effect and making the patient susceptible to recurrences of CAD.

**Figure 1:**
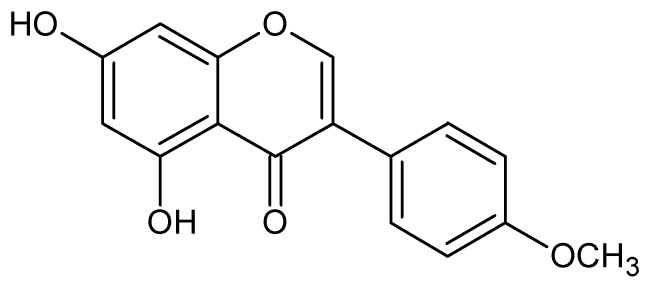
Chemical structure of biochanin A (5,7-dihydroxy-4‘-methoxy isoflavone).

Because of resistance and negative side effects, alternatives to ATB therapy for microbial pathogens have been searched for among either other microorganisms [22] or plant-derived products [27,28]. *In vitro* studies have result in the finding that several flavonoids, phenolic secondary metabolites of plants, possess strong antimicrobial activity [28,29] with Gram-positive bacteria being the most sensitive germs [28]. Isoflavonoids, a subgroup of flavonoids with phyteostrogenic activity, are widely reported to function within the antimicrobial protection, because they act as phytoalexins [30,31], suppress a wide range of both Gram-negative and Gram-positive bacteria strains [32,33,34,35,36] and possess antifungal activity [37,38,39]. The strongest antimicrobial action has been observed for isoflavonoids from *Erythrina poeppigiana* [36] and genistein [34,35] against staphylococci and sophoraisoflavone A and 6,8-diprenylgenistein against *Staphylococcus aureus*, salmonellae, shigellae and vibria [32]. Biochanin A (Figure 1), an isoflavonoid occurring commonly in plants, shows strong antifungal activity [37,38,39] and inhibits among others [32] the growth of *Mycobacterium smegmatis*, a bacterium used for the research of other mycobacteria (especially *M. tuberculosis*) *in vitro* [40]. Because of its previously described ability to suppress the growth of various microorganisms and our preliminary results from ample investigation on *in vitro* antimicrobial activity of 9 isoflavones (daidzein, genistein, glycitin, biochanin A, formononetin, prunetin, genistin, daidzin and coumestrol) that suggests its selective antibacterial action [41], the focus of this study is to evaluate the antibacterial effect of biochanin A on the survival and growth of opportunistic gut microflora represented by potentially pathogenic clostridia and beneficial bifidobacteria.

## 2. Results and Discussion

In this study, biochanin A possessed a selective growth inhibitory effect against type cultures and wild isolates of genera *Clostridium* and *Bifidobacterium*, both usually regarded as Gram-positive, anaerobic rods [23,42] belonging to natural gut microflora. It was found that biochanin A inhibited all clostridia tested; however, its effectiveness differs considerably depending upon the concentration used. The results summarized in Table 1 show that the most susceptible strains with MIC 64 μg/mL were the type culture as well as the clinical isolates of *Cl. clostridioforme*, a relatively antimicrobial resistant anaerobe that has been involved in a variety of infections, including bacteraemia [43]. The inhibition activity at MIC level 256 μg/mL was observed for type cultures of *Cl. ramosum* and *Cl. paraputrificum* and wild isolates of *Cl. ramosum* and *Cl. difficile*, microorganisms responsible for severe secondary infections especially in children [44]. Additionally *Cl. difficile* is the most studied and widespread species of all clostridia, with an alarmingly harmful incidence among hospitalized patients. Among clinical isolates, *Cl. acetobutylicum* and *Cl. butylicum* were moderately susceptible with MIC 512 μg/mL, which is rather positive because both these microorganisms usually do not cause infections either in humans or in animals; conversely they are producers of several important organic chemicals. The same level of inhibition (MIC 512 μg/mL) was assayed for type strains of *Cl. butyricum* and *Cl. tertium*, where the last mentioned causes more accurately exogenous infections. The most resistant of all clostridia was the type culture of *Cl. perfringens*, which was inhibited at MIC levels as high as 1,024 μg/mL.

In contrast to all clostridia tested, biochanin A had no effect on the growth of any strain of bifidobacteria at a concentration of 4,096 μg/mL, suggesting that MIC towards beneficial microbiota is at least four times higher than the level of the highest clostridia inhibition presented in this paper. MIC values of the broad-spectrum ATB tetracycline were assayed simultaneously as a reference, because it is used in the treatment of clostridial infections with a harmful influence on a wide range of *Bifidobacterium* strains [45,46]. In this study, an inhibitory effect of tetracycline was observed from 0.0156 to 8 μg/mL towards clostridia (Table 1), but its MICs on tested bifidobacteria already started at 0.5 μg/mL and did not exceed the level of 1 μg/mL. Accordingly, pathogenic clostridia were inhibited by MICs averaging higher (MIC 1.22 μg/mL) than the representatives of positive gut microflora (MIC 0.83 μg/mL), which indicates that tetracycline may have a negative impact on the balance in the intestinal microecosystem, resulting in additional disorders.

Biochanin A has the ability to inhibit a number of microorganisms, above all fungi [37,38,39]. Among bacteria it moderately restrains *S. aureus*, *Salmonella* spp., *Shigella* spp., *Klebsiella* spp., *Pseudomonas* spp., *Vibrio cholerae* and *V. parahaemolyticus* when tested at concentrations ranging from 25 to 200 μg/mL [32] and suppresses *Mycobacterium smegmatis*, where MIC was assessed as 256 μg/mL [40]. Moreover, besides its direct inhibitory effect on microorganisms, biochanin A also has the ability to potentiate the antimicrobial action of other compounds. For example, Morel *et al*. [47] found that 6.25 μg/mL of biochanin A in combination with 30 μg/mL of berberine or 30 μg/mL of α-linolenic acid completely inhibited *S. aureus* and *Berberis megaterium* growth, whilst separately concentrations of each compound were subinhibitory. To our best knowledge, no study describing the inhibitory action of biochanin A or other isoflavonoids towards clostridia is available at the moment. However, despite the fact that the MICs presented in this paper are rather high in comparison with conventional antibiotics, and the most effective isoflavonoids (the MIC of isolupalbigenin against methicillin-resistant *S. aureus* was assayed to range from 1.56 to 3.13 μg/mL depending on the strain [48]), the potential for practical use of biochanin A is supported by the fact that this compound is widely present in various types of common edible plants, including fodder for animals. Biochanin A is found mainly in *Trifolium pratense* L., where its average concentration ranges 1.55–14.59 mg g^−1^ dry weight [49]. How is the amount of biochanin A expressed in the intestinal tract of warm-blooded consumers has been investigated *in vivo* on animal models (sheep), where Batterham *et al*. [50] found the concentration of orally administrated biochanin A in rumen liquor decreased by 10, 50 and 70% in 1.5, 4 and 6 hours after dosing, respectively; and Davies *et al*. [51] also observed the ratio of biochanin A and its metabolite genistein in time. There is no study on biochanin A content in bowel in humans. As a common plant product, biochanin A (as well as other isoflavonoids) is generally considered to be non-toxic. Contrarily, it A protects human cortical neurons from glutamate toxicity together with other isoflavones from *Trifolium pratense* L. (Red Clover) [52]. Therefore, it is supposed first and foremost to exert its biological and pharmacological effects, making it a potentially important molecule for dietary pharmaceutical uses.

Although clostridia are generally considered to be Gram-positive, this genus is not uniform in its Gram-staining. According to Yuli *et al.* [43] *Cl. clostridioforme* may stand as Gram-negative; *Cl. paraputrificum* is Gram-positive [53], but can be rapidly turned to Gram-negative [54] and *Cl. ramosum* has rather variable Gram-stain [7]. In a previous study on antimicrobial action of isoflavonoids, Gnanamanickan and Smith [33] found isoflavonoids to be selectively toxic to Gram-positive bacteria. In this study, biochanin A inhibited several but not all Gram-variable bacteria (*Cl. clostridioforme*, *Cl. ramosum*), suppressing the growth of Gram-positive *Cl. difficile* and *Cl. paraputrificum* while not restraining the growth of any Gram-positive bifidobacteria, which is in line with the findings of a recent study published by Hong *et al.* [34] for the structurally closely related isoflavonoid genistein, suggesting that antimicrobial activity of biochanin A is independent of the bacterial Gram-staining characteristics.

In general, flavonoids are considered to profit from their ability to complex with extracellular and soluble proteins and to complex with bacterial cell walls or to disrupt microbial membranes [55,56], and cell-wall structure is considered to be an important factor in this mechanism. According to our results, this behaviour may not be inferred for biochanin A because of its selective inhibition of Gram-variable bacteria. Therefore, it may be presuppose that the mode of action of biochanin A is not directly connected with the cell walls. Since genistein, which except for one surplus methyl group at position 4’ has a molecule structure fairly similar to biochanin A, is believed to bear its antimicrobial activity from the ability to inhibit DNA topoisomerases that participate in various aspects of DNA metabolism, the interference related metabolic pathways may be responsible for the antimicrobial action of biochanin A. A structure-activity assay of growth-inhibiting activity of biochanin A has not been made, but Lee considered the presence of methoxyl group in molecule to be essential for antimicrobial activity as well as the presence of a hydroxyl group at position 5 [57], which is incident in the structure of biochanin A. Mechanism of selective inhibition has not been investigated in this study; nevertheless, it can be assumed that it may be based either on DNA topoisomerases inhibition (like as genistein) or inhibition of other biosynthetic pathway in the cell. Therefore, further research is necessary to explain the selectivity and to establish whether this activity is exerted *in vivo* after oral administration of biochanin A by consumers.

## 3. Experimental

### 3.1. Microbial cultures and media

Bacterial strains were obtained from the American Type Culture Collection (ATCC, Manassas, VA, USA), from the Czech Collection of Microorganisms (CCM, Faculty of Science, Masaryk University, Brno, Czech Republic) and from the German Resource Centre for Biological Material (DSMZ, Braunschweig, Germany). Five clostridial strains were isolated from infant faeces using Clostridial reinforced agar (Oxoid, Basingstoke, UK). Pure cultures were identified to the genus level using a fluorescence *in situ* hybridization kit with a *Clostridium butyricum* group-specific probe (Ribotechnologie, NL). Isolates were characterized using ANAEROtest 23 (Lachema, Czech Republic) and API 20A (BioMerieux, France). The effectiveness of biochanin A was evaluated in six strains of *Bifidobacterium* spp. and nine strains of *Clostridium* spp (Table 1). *Bifidobacteria* were grown and tested in Trypticase-Peptone-Yeast extract (TPY medium), *Clostridia* in Wilkins-Chalgren anaerobe broth, both under anaerobic conditions using Anaerobic Jar HP11; both the media and the anaerobic jar were purchased from Oxoid.

### 3.2. Antimicrobial assay

The antimicrobial activity was evaluated in *in vitro* conditions by the broth microdilution method [58] using 96-well microtiter plates. Initially, biochanin A (Sigma-Aldrich, Prague, Czech Republic) was diluted in dimethyl sulfoxide (DMSO, Lach-Ner, Czech Republic) creating the stock solution and afterwards in appropriate broth media for the final concentration 1% of DMSO. The samples were tested starting from concentration 4,096 μg/mL for bifidobacteria and 1,024 μg/mL for clostridia in double fold dilutions (eight) for each bacterium. Each well of the microtiter plate was inoculated with a bacterial suspension (10 µL) at a density of 10^7^ colony-forming units (CFU)/mL, incubated at 37 °C for 48 h, and then observed for minimum inhibitory concentration (MIC). The growth of microorganisms was determined spectrophotometrically as turbidity using Multiscan Ascent Microplate reader (Thermo Fisher Scientific, Waltham, MA, USA) at 405 nm. The MIC was determined as the lowest dilution that resulted in an 80% reduction in growth compared with the biochanin A-free growth control. The solution of DMSO (1% v/v) was simultaneously assayed as the negative control. The susceptibility of all microorganisms to tetracycline (Sigma-Aldrich, Prague, Czech Republic) was evaluated as a positive control. The tests were performed as three independent experiments, each carried in triplicate.

## 4. Conclusions

In conclusion, the present study has demonstrated that biochanin A has a selective action against certain enteropathogenic microorganisms and do not suppress beneficial bowel microflora. Regarding its non-toxic nature and specific activity, the use of biochanin A (e.g. in combination with probiotics) may augment the effectiveness of antimicrobial therapies and reform or balance the health of gut microflora. However, before starting the use of biochanin A for natural phytochemical food supplements or pharmaceuticals, investigations regarding the exact mechanism of action should be performed.

## Figures and Tables

**Table 1:** The selective antimicrobial effect of biochanin A *in vitro* against bifidobacteria and clostridia.

Bacterial strains	MIC^1^ (μg/mL)	
	biochanin A	tetracycline
*Bifidobacterium* spp.		
*B. animalis* CCM^2^ 4988	>4096	1
*B. bifidum* ATCC^3^ 29 521	>4096	1
*B. breve* ATCC 15 700	>4096	0.5
*B. catenulatum* CCM 4989	>4096	0.5
*B. infantis* ATCC 17 930	>4096	1
*B. longum* ATCC 15 707	>4096	1
Average	>4096	0.83
*Clostridium* spp.		
*Cl. butyricum* DSM^4^ 10702	512	0.0625
*Cl. clostridioforme* DSM 933	64	0.025
*Cl. paraputrificum* DSM 2630	256	8
*Cl. perfringens* DSM 11778	1024	4
*Cl. ramosum* DSM 1402	256	1
*Cl. tertium* DSM 2485	512	0.0625
*Cl. acetobutylicum* I^5^1	512	0.0625
*Cl. butylicum* I 2	512	0.0625
*Cl. clostridioforme* I 3	64	0.0156
*Cl. difficile* I 4	256	0.0625
*Cl. ramosum* I 5	256	0.0625
Average	384	1.2196

^1^ MIC - minimum inhibitory concentration; ^2^ CCM - Czech Collection of Microorganisms; ^3^ ATCC - American Type Culture Collection; ^4^ DSM - German Resource Centre for Biological Material; ^5^ I - Isolated from infant faeces.
